# Supplementary material for: Inhibitory Effect of Avenanthramides (Avn) on Tyrosinase Activity and Melanogenesis in α-MSH-Activated SK-MEL-2 Cells: In Vitro and In Silico Analysis
Source: Int J Mol Sci. 2021 Jul 22;22(15):7814. doi: 10.3390/ijms22157814 (PMC8345984; doi:10.3390/ijms22157814)
Supplement: Supplementary file 1 [file ijms-22-07814-s001.zip › ijms-1275226-supplementary.pdf]

Supplementary Figures

Inhibitory effect of Avn-A-B-C on tyrosinase activity and melanogenesis in α-MSH-activated SK-MEL-2 cells: in vitro and in silico analysis

Junyoung Park[1#], Hyunju Choi[1#], Tamina Park[2,3], Moon-Jo Lee[4], Hak-Seong Lim[1], Woong-Suk Yang[5], Cher-Won Hwang[6], Daeui Park[2,3] and Cheorl-Ho Kim[1*]*

1Molecular and Cellular Glycobiology Unit, Department of Biological Sciences, SungKyunKwan University, 300 Chunchun-Dong, Jangan-Gu, Suwon City, Kyunggi-Do 440-746, Korea.

2Department of Predictive Toxicology, Korea Institute of Toxicology, Daejeon 34114, Korea

3Department of Human and Environmental Toxicology, University of Science and Technology, Daejeon 34113, Korea.

4Department of Herb Science, Dong-Eui Institute of Technology, Busan, Republic of Korea.

5Nodaji Co., Ltd., Pohang, Gyeongbuk, Republic of Korea.

6Department of AGEE, Handong University, Pohang, Gyeongbuk, Republic of Korea.

Derek Nexus Report

Report Information

Author	Report date	Prediction date	Program version
Admin	01 July 2021 13:51:06	01 July 2021 13:51:05	Derek Nexus: 6.1.0, Nexus: 2.3.0

Processing Options

**Selected Species** mammal	**Selected Knowledge Base(s)** Derek KB 2020 1.0	**Reasoning Level** At least EQUIVOCAL
**Perceive tautomers** Yes	**Perceive mixtures** Yes	**Match alerts without rules** No
**Show Open likelihood** No	**Show Negative Predictions** Yes	**Show Rapid Prototypes** Yes
**Filter nearest neighbours on misclassified features** Yes		

Submitted Compound - AvnA.mol

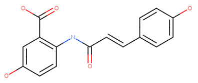

Smiles: C1=C(C=CC(=C1C(=O)O)NC(=O)C=CC2=CC=C(C=C2)O)O

Exact Mol Mass 299.0794
Average Mol Mass 299.28 (Source: Lhasa Limited, version 1.0)
Log Kp -2.48 (Source: Potts & Guy, version 1.0)
Log P 2.91 (Source: BioByte Corp., version 5.9)

Predictions

Knowledge Base: Derek KB 2020 1.0

Version	Last Modified Date	Certified by
1.0	26/03/2020 17:28:54	Lhasa Limited, Leeds, Yorkshire, UK

Reasoning Summary

◆ **Hepatotoxicity in mammal is PLAUSIBLE**
- Alert matched: 619 para-Aminophenol or derivative

◆ **Mitochondrial dysfunction in mammal is EQUIVOCAL**
- Alert matched: RapidPrototype111 para-Aminophenol or derivative

◆ **Nephrotoxicity in mammal is PLAUSIBLE**
- Alert matched: 818 para-Aminophenol or derivative

◆ **Skin sensitisation in mammal is EQUIVOCAL**
- Alert matched: 439 Substituted phenol

Endpoints not firing any alerts at the selected reasoning level (57)

5alpha-Reductase inhibition	Lachrymation
Adrenal gland toxicity	Methaemoglobinaemia
alpha-2-mu-Globulin nephropathy	Mutagenicity in vitro
Anaphylaxis	Mutagenicity in vivo
Androgen receptor modulation	Neurotoxicity
Bladder disorders	Non-specific genotoxicity in vitro
Bladder urothelial hyperplasia	Non-specific genotoxicity in vivo
Blood in urine	Occupational asthma
Bone marrow toxicity	Ocular toxicity
Bradycardia	Oestrogen receptor modulation
Carcinogenicity	Oestrogenicity
Cardiotoxicity	Peroxisome proliferation
Cerebral oedema	Phospholipidosis
Chloracne	Photo-induced chromosome damage in vitro
Cholinesterase inhibition	Photo-induced non-specific genotoxicity in vitro
Chromosome damage in vitro	Photo-induced non-specific genotoxicity in vivo
Chromosome damage in vivo	Photoallergenicity
Cumulative effect on white cell count and immunology	Photocarcinogenicity
Cyanide-type effects	Photomutagenicity in vitro
Developmental toxicity	Phototoxicity
Glucocorticoid receptor agonism	Pulmonary toxicity
HERG channel inhibition in vitro	Respiratory sensitisation
High acute toxicity	Splenotoxicity
Irritation (of the eye)	Teratogenicity
Irritation (of the gastrointestinal tract)	Testicular toxicity
Irritation (of the respiratory tract)	Thyroid toxicity
Irritation (of the skin)	Uncoupler of oxidative phosphorylation
Kidney disorders	Urolithiasis

Alert Descriptions

! Alert: 439 Substituted phenol (from KB: Derek KB 2020 1.0)

Alert Description Image

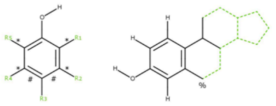

R1 = H, CH, F, Cl, Br, I
R2, R4 = H, F, Cl, Br, I, CH2 or C (sp2 or sp hybridised) except C(=O)OR6
R3, R5 = H, F, Cl, Br, I, C except C(=O)OR7
R1-R5 cannot all be hydrogen
R6, R7 = C, H

Atoms marked % require a double bond and cannot be attached to additional heteroatoms
All ring fusions allowed except bonds marked # cannot be fusion bonds
Bonds marked * can be single or part of an aromatic fusion
Dashed bonds can be either single or double

Match with query compound

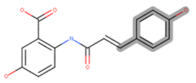

Supplementary Data Fig. 1. QSAR Prediction results for AvnA by the Derek

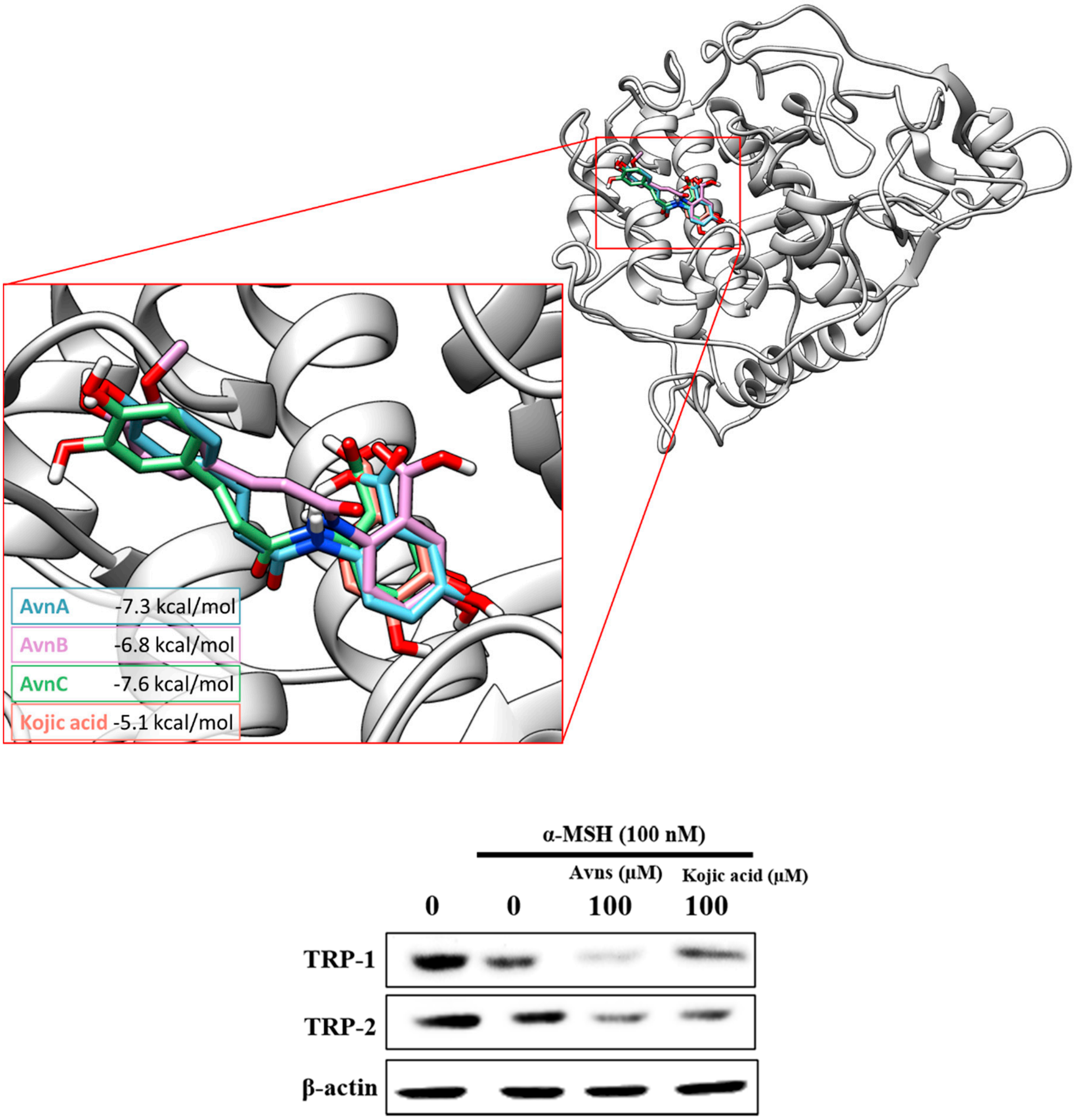

Supplementary Data Fig. 2. Positive control experiments
